# Immunogenicity of propagation-restricted vesicular stomatitis virus encoding Ebola virus glycoprotein in guinea pigs

**DOI:** 10.1099/jgv.0.001085

**Published:** 2018-06-05

**Authors:** Samira Locher, Marc Schweneker, Jürgen Hausmann, Gert Zimmer

**Affiliations:** ^1^​ Institut für Virologie und Immunologie (IVI), Sensemattstrasse 293, CH-3147 Mittelhäusern, Switzerland; ^2^​ Bavarian Nordic GmbH, Fraunhoferstraße 13, D-82152 Martinsried, Germany

**Keywords:** vesicular stomatitis virus, Ebola virus, vector vaccine, biosafety, viral glycoprotein, reversion, vaccinia virus, neutralizing antibody

## Abstract

Vesicular stomatitis virus (VSV) expressing the Ebola virus (EBOV) glycoprotein (GP) in place of the VSV glycoprotein G (VSV/EBOV-GP) is a promising EBOV vaccine candidate which has already entered clinical phase 3 studies. Although this chimeric virus was tolerated overall by volunteers, it still caused viremia and adverse effects such as fever and arthritis, suggesting that it might not be sufficiently attenuated. In this study, the VSV/EBOV-GP vector was further modified in order to achieve attenuation while maintaining immunogenicity. All recombinant VSV constructs were propagated on VSV G protein expressing helper cells and used to immunize guinea pigs via the intramuscular route. The humoral immune response was analysed by EBOV-GP-specific fluorescence-linked immunosorbent assay, plaque reduction neutralization test and *in vitro* virus-spreading inhibition test that employed recombinant VSV/EBOV-GP expressing either green fluorescent protein or secreted Nano luciferase. Most modified vector constructs induced lower levels of protective antibodies than the parental VSV/EBOV-GP or a recombinant modified vaccinia virus Ankara vector encoding full-length EBOV-GP. However, the VSV/EBOV-GP(F88A) mutant was at least as immunogenic as the parental vaccine virus although it was highly propagation-restricted. This finding suggests that VSV-vectored vaccines need not be propagation-competent to induce a robust humoral immune response. However, VSV/EBOV-GP(F88A) rapidly reverted to a fully propagation-competent virus indicating that a single-point mutation is not sufficient to maintain the propagation-restricted phenotype.

## Introduction

Since the first isolation of Marburg virus in 1967, several other filoviruses have been discovered. The family of *Filoviridae* presently comprises the genus *Marburgvirus* with the species *Marburg virus* (MARV) and *Ravn virus* (RAVV), the genus *Ebolavirus* containing the species *Zaire ebolavirus* (EBOV), *Sudan ebolavirus* (SUDV), *Bundibugyo ebolavirus* (BDBV), *Reston ebolavirus* (RESTV), and *Taï Forest ebolavirus* (TAFV), and the genus *Cuevavirus* with the species *Lloviu virus* (LLOV). Several filoviruses cause severe haemorrhagic fever diseases in humans and non-human primates with the highest mortality rates associated with *Zaire ebolavirus*. The first EBOV outbreak was noted in 1976 in the Democratic Republic of Congo (former Zaire). Since then several small sporadic outbreaks with a limited number of persons affected have occurred [1]. An unusually large outbreak took place in 2014 in West Africa and caused at least 28 637 cases of Ebola virus disease (EVD), claiming 11 315 deaths [2, 3]. This outbreak has greatly pushed the search for vaccines and antivirals which would protect from this fatal disease. However, all these efforts were complicated by the absolute necessity to handle EBOV and other filoviruses in laboratories strictly complying with biosafety level 4.

A recombinant vesicular stomatitis virus (VSV) expressing the EBOV glycoprotein (EBOV-GP) in place of the VSV glycoprotein (VSV-G) was one of the first Ebola vaccine candidates showing promising results. This live-attenuated recombinant vector vaccine induced a protective immune response in non-human primates [4] and mediated protection even if applied post exposure to EBOV [5]. There is strong evidence that protection was mediated by antibodies directed to the EBOV glycoprotein [6]. As wild-type VSV is characterized by a pronounced neurotropism in rodent animal models [7, 8], concerns over the safety of the chimeric VSV/EBOV-GP vaccine were raised. However, the chimeric virus was demonstrated to completely lack neurovirulence in non-human primates [9] and was even tolerated by immunocompromised animals [10]. Following the EBOV outbreak in West Africa in 2014 clinical phase I/II studies were launched in order to validate the recombinant VSV vector vaccine in human volunteers. The vaccine seemed to induce a protective immune response [11]. However, adverse effects such as fever and long-lasting arthritis were observed in some volunteers [12, 13], suggesting that the VSV vector might not be sufficiently attenuated.

Since expression of EBOV-GP in place of VSV-G was found to attenuate the VSV vector to a significant degree, no additional mutations have been introduced into the vector backbone. In this study, we aimed at further modifying the VSV vector backbone or the EBOV-GP antigen in order to produce a more attenuated but still immunogenic vaccine. The humoral immune response to these experimental vaccines was studied in the guinea pig model by employing EBOV-GP-specific fluorescence-linked immunosorbent assay (FLISA), plaque reduction neutralization test (PRNT) and a novel virus-spreading inhibition test taking advantage of recombinant VSV/EBOV-GP reporter virus-encoding secreted Nano luciferase (sNLuc). The attenuated vector vaccines were compared with the unmodified VSV*ΔG(EBOV-GP) vaccine and with recombinant modified vaccinia virus Ankara (MVA) vectors expressing the same EBOV-GP antigen.

## Results

### Generation of modified VSV-EBOV vaccine vectors

**Fig. 1.**
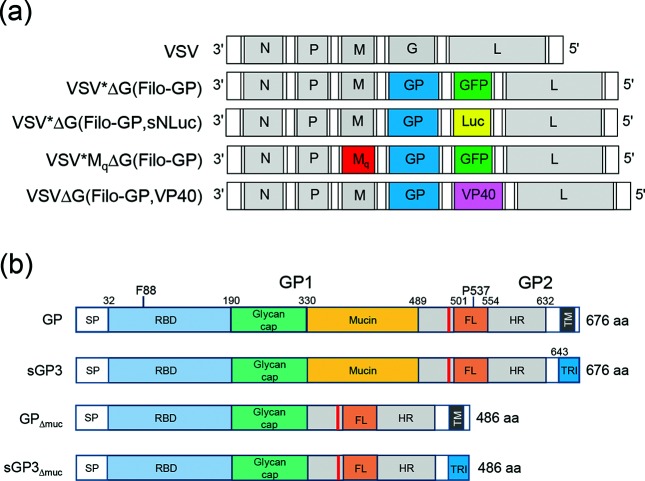
Genome maps of recombinant VSV vectors. (a) The original VSV contains five transcription units encoding the nucleoprotein N, the phosphoprotein P, the matrix protein M, the glycoprotein G and the large RNA-dependent RNA polymerase L. The VSV vector was modified by replacing the G gene with the EBOV-GP gene (either authentic or modified) and by inserting an additional transcription unit encoding either GFP, sNLuc or EBOV-VP40. Mq denotes a modified M gene encoding a mutant M protein which is characterized by the amino acid changes M33A, M51R, V221F and S226R. (b) Protein maps of authentic and modified EBOV-GP, indicating the location of the signal peptide (SP), receptor-binding domain (RBD), glycan cap, mucin-like domain (mucin), fusion loop (FL), heptad repeat (HR) region and transmembrane (TM) domain. The furin cleavage site (red line), the cleavage products GP1 and GP2, and the amino acid positions F88 and P537 are indicated as well.

The EBOV vaccine candidate which has already entered clinical phase 3 studies represents a chimeric VSV in which the envelope glycoprotein (G) gene has been replaced by the EBOV (species Zaire) glycoprotein (GP) gene [14]. We generated a very similar virus, VSV*ΔG(EBOV-GP), which differed from VSV/EBOV-GP in additionally encoding a reporter protein, either green fluorescent protein (GFP) or sNLuc (Fig. 1a). The EBOV-GP contains a heavily O-glycosylated mucin-like domain which may have an impact on the immunogenicity and cytotoxicity of the protein [15, 16]. To analyse the role of this domain in VSV vector-driven immune responses, recombinant VSV with a modified EBOV-GP lacking the mucin-like domain (VSV*ΔG(EBOV-GP_Δmuc_) was generated (Fig. 1b). In addition, the recombinant vector vaccine VSVΔG(EBOV-GP,VP40) encoding both EBOV-GP and EBOV-VP40 was produced (Fig. 1a), as previous results suggested a positive impact of the EBOV matrix protein VP40 on vaccine efficacy [17].

Two strategies were pursued to make the VSV vector safer – modification of the vector backbone and modification of the EBOV-GP antigen. The vector backbone was modified by introducing four mutations into the matrix (M) protein gene (Mq) that are known to abolish the host shut-off activity of the protein [18]. We anticipated that the resulting vector, VSV*MqΔG(EBOV-GP) (Fig. 1a), would be unable to block the synthesis and release of type I IFN, which would interfere with the dissemination of the viral vector. The EBOV-GP antigen was modified in order to produce a propagation-incompetent VSV-vectored vaccine. We hypothesized that a EBOV-GP lacking the transmembrane domain would not be incorporated into the VSV envelope and thus could not substitute for the deleted VSV-G protein. Therefore, a soluble version of EBOV-GP, consisting of the GP ectodomain and a carboxyterminal GCN4-pII trimerization domain (Fig. 1b), was expressed from the VSV*ΔG(EBOV-sGP3) genome. In addition, VSV*ΔG(EBOV-sGP3_Δmuc_) was constructed which encoded a secreted trimeric version of the glycoprotein without the mucin-like domain (Fig. 1b). As an alternative approach, full-length but functionally impaired EBOV-GP with point mutations F88A or P537R were expressed from the VSVΔG genome. The mutation F88A has previously been shown to render the glycoprotein defective for entry into a variety of human cell types [19, 20]. The mutation P537R is located in the putative fusion domain and was demonstrated to interfere with the membrane fusion activity of EBOV-GP [21, 22].

### Analysis of recombinant VSV vector replication *in vitro*

**Fig. 2.**
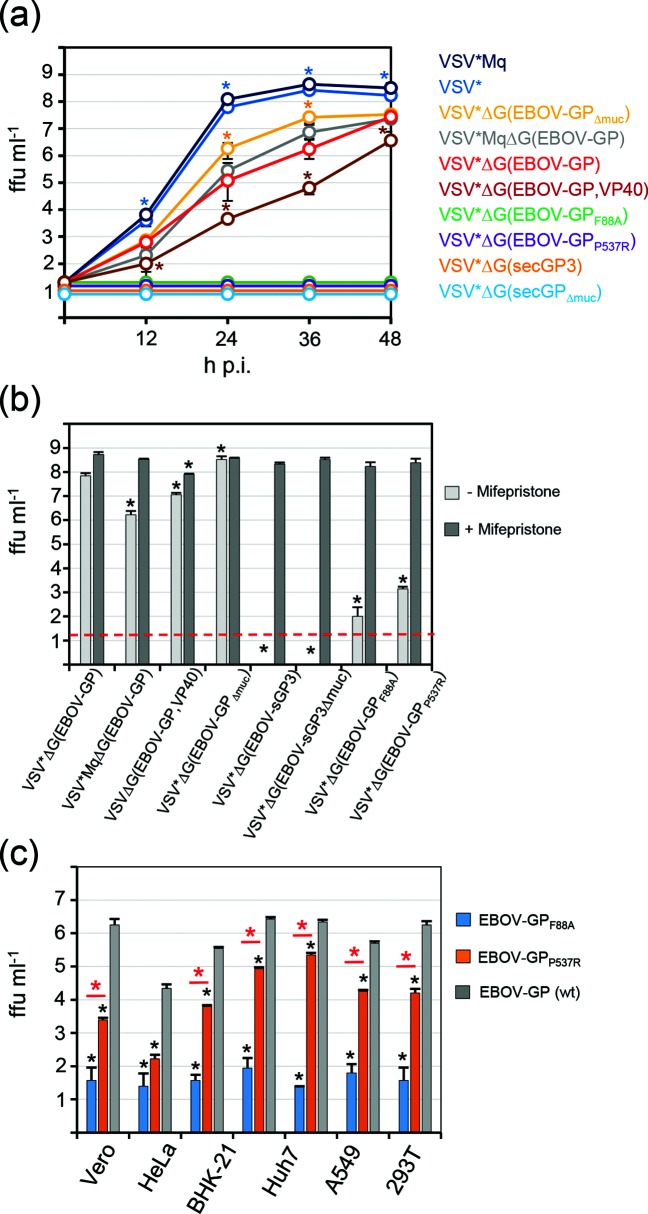
Propagation competence of recombinant VSV vectors. (a) Multi-cycle replication of recombinant VSV. Vero cells grown in six-well plates were infected with the indicated recombinant viruses using an m.o.i. of 0.0001 ffu cell^−1^. At the indicated times, aliquots of the cell-culture supernatant were collected and infectious virus titrated on Vero cells. Mean values and sd of three independent experiments are shown. Asterisks indicate significantly different infectious virus titres when compared to VSV*ΔG(EBOV-GP). (b) Virus yield on helper cells. BHK-G43 cells in 24-well plates were treated with mifepristone to induce VSV-G protein expression (dark grey bars) or were left untreated (light grey bars). Cells were infected with the indicated viruses using an m.o.i. of 0.1 ffu cell^−1^ and maintained for 24 h in medium with or without mifepristone. Medium without mifepristone was supplemented with a neutralizing antibody directed to the VSV-G protein in order to inactivate any remaining input virus. Infectious virus released into the cell-culture medium was titrated on Vero cells. Results are shown as the mean plus sd of three independent experiments. Asterisks indicate significantly different infectious virus titres when compared to VSV*ΔG(EBOV-GP). (c) Propagation of GP mutant VSV on mammalian cell lines. The indicated cell lines were infected with VSV*ΔG(EBOV-GP_F88A_) (blue bars), VSV*ΔG(EBOV-GP_P537R_) (orange bars) and parental VSV*ΔG(EBOV-GP) (gey bars) using an m.o.i. of 0.1 ffu cell^−1^ and incubated for 24 h in the presence of neutralizing antibody directed to the VSV-G protein. Infectious virus titres released into the cell-culture supernatant were determined. Mean titres and sd of three infection experiments are shown. Black asterisks indicate significantly different infectious virus titres when compared to VSV*ΔG(EBOV-GP). Red asterisks indicate significantly different virus titres when comparing VSV*ΔG(EBOV-GP_F88A_) with VSV*ΔG(EBOV-GP_P537R_).

**Fig. 3.**
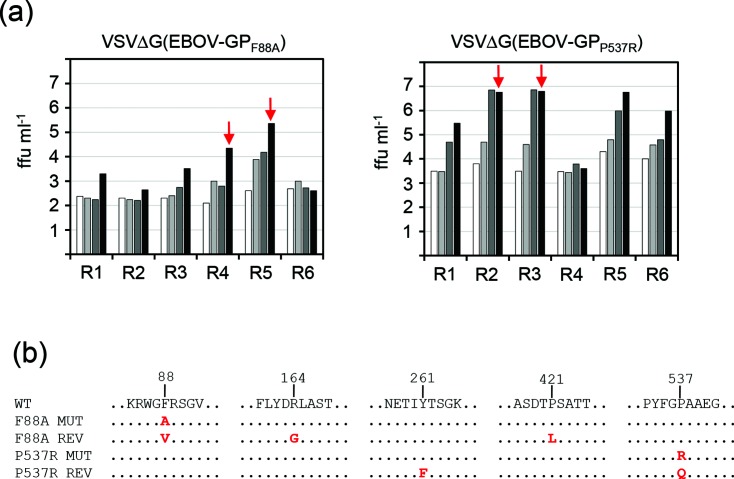
Reversion of the growth-restricted phenotype of VSV*ΔG(EBOV-GP_F88A_) and VSV*ΔG(EBOV-GP_P537R_). (a) The indicated viruses were serially passaged on BHK-21 cells (replicates R1 to R6) and infectious virus titres (24 h p.i.) determined for passage 2 (white bars), passage 3 (light grey bars), passage 4 (dark grey bars) and passage 5 (black bars). Passage 5 viruses that were used to determine the cDNA sequence of GP are indicated by red arrows. (b) The complete primary sequence of GP from passaged viruses was determined but only regions containing amino acid changes are depicted. The same mutations were found in the second replicate of VSV*ΔG(EBOV-GP_F88A_) and VSV*ΔG(EBOV-GP_P537R_), respectively.

Multi-step replication of the generated recombinant VSV vectors was analysed on Vero cells using a m.o.i. of 0.0001 focus-forming units (ffu) cell^−1^ (Fig. 2a). The reference virus VSV*ΔG(EBOV-GP) (red curve) reached infectious titres of 2.7×10^7^ ffu ml^−1^ at 48 h post infection (p.i.) while the parental virus VSV* encoding the homotypic G protein replicated faster and produced significantly higher titres at all times of the kinetics (light blue curve). A maximum infectious titre of 5×10^8^ ffu ml^−1^ was reached by VSV* at 36 h p.i. These findings confirmed the previous notion that recombinant VSV encoding the EBOV-GP in place of the VSV-G envelope protein is attenuated compared to wild-type VSV, although it is still able to produce significantly high infectious titres in cell culture [14]. The replication kinetics of the modified vector VSV*MqΔG(EBOV-GP) encoding the mutant matrix protein Mq (grey curve) did not reveal significant differences when compared with the VSV*ΔG(EBOV-GP) kinetics. Similarly, the corresponding parental virus VSV*Mq encoding the homotypic VSV-G glycoprotein showed a very similar (not statistically different) kinetics as VSV*, indicating that the Mq protein did not negatively affect viral replication in this cell line. Compared to the reference virus VSV*ΔG(EBOV-GP), VSV*ΔG(EBOV-GP_Δmuc_), which lacked the mucin-like domain of EBOV-GP, produced significantly higher titres at 24 and 36 h p.i. (yellow curve). In contrast, VSVΔG(EBOV-GP,VP40) was significantly attenuated compared to VSV*ΔG(EBOV-GP) and reached only 3.6×10^6^ ffu ml^−1^ at 48 h p.i. (brown curve). As expected, the recombinant viruses encoding soluble EBOV-GP, VSV*ΔG(EBOV-sGP3) and VSV*ΔG(EBOV-sGP3_Δmuc_), were not able to propagate on Vero cells (Fig. 2a). Similarly, chimeric VSV containing full-length GP with either the mutation F88A or P537R did not produce significant levels of infectious virus following infection at low dose (m.o.i. of 0.0001 ffu cell^−1^). However, all viruses unable to replicate autonomously on Vero cells could be propagated to high titres (about 10^8^ ffu ml^−1^) on BHK-G43 helper cells expressing the VSV-G protein in a regulated manner (Fig. 2b). Using an m.o.i. of 0.1 ffu cell^−1^, VSV*ΔG(EBOV-GP_F88A_) and VSV*ΔG(EBOV-GP_P537R_) produced low infectious virus titres on non-induced BHK-G43 helper cells (about 10^2^ and 10^3^ ffu ml^−1^, respectively). Compared to the reference virus VSV*ΔG(EBOV-GP), propagation of VSV*ΔG(EBOV-GP_F88A_) and VSV*ΔG(EBOV-GP_P537R_) was significantly restricted on other cell lines (Fig. 2c). However, VSV*ΔG(EBOV-GP_P537R_) turned out to be less restricted than VSV*ΔG(EBOV-GP_F88A_) on most cell lines with the exception of HeLa cells. In particular, Huh7 cells allowed VSV*ΔG(EBOV-GP_P537R_) to replicate to titres that were only 1 log_10_ lower than those produced by the parental virus VSV*ΔG(EBOV-GP). In order to elucidate the stability of the attenuated phenotypes, VSV*ΔG(EBOV-GP_F88A_) and VSV*ΔG(EBOV-GP_P537R_) were serially passaged on BHK-21 cells, each virus in six replicates (Fig. 3a). After a few passages, viruses producing significantly higher titres than the original viruses emerged in several replicates. Sequence analysis of the GP cDNA derived from two selected passage 5 viruses revealed three mutations in the GP_F88A_ gene, A88V, R164G and P421L, and two mutations in the GP_P537R_ gene, Y261F and R537Q (Fig. 3b), indicating that the propagation-restricted viruses VSV*ΔG(EBOV-GP_F88A_) and VSV*ΔG(EBOV-GP_P537R_) are not genetically stable.

**Fig. 4.**
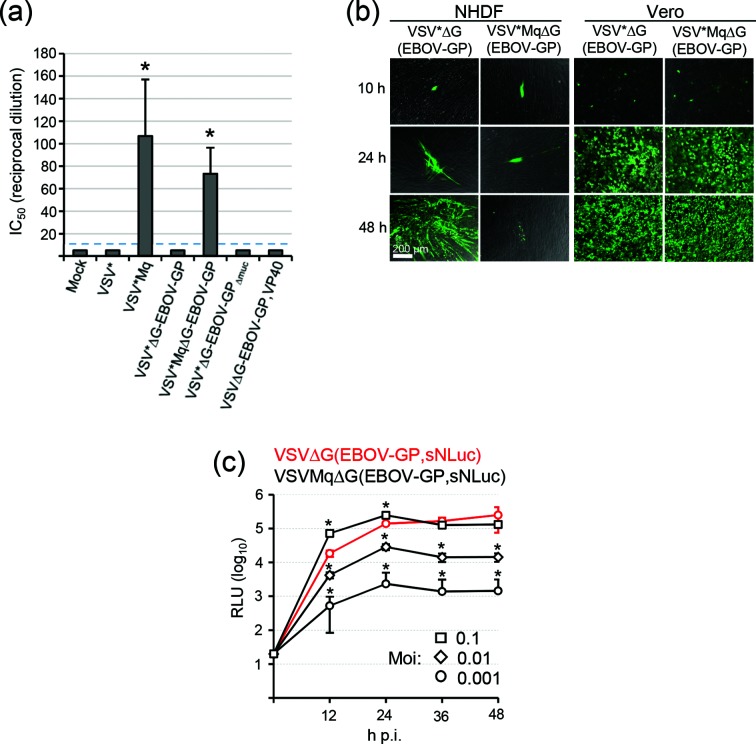
Induction of type I IFN synthesis in VSV vector-infected cells. (a) Release of type I IFN by infected cells. NHDF were infected with the indicated viruses (m.o.i. of 10). At 24 h p.i., cell-culture supernatants were sampled and heated for 30 min at 55 °C to inactivate infectious virus. The antiviral activity released into the cell-culture medium was titrated on HeLa cells using a previously described VSV replicon-based bioassay [24]. The antiviral activity was expressed as inhibitory concentration 50 % (IC_50_). Mean values and sd of three infection experiments are shown. The broken line indicates the lower limit of detection. Asterisks indicate secretion of type I IFN at levels that are significantly different (*P*<0.05) from the mock control. (b) Spreading of chimeric VSV in cell culture. NHDF and Vero cells were infected with either VSV*∆G(EBOV-GP) or VSV*Mq∆G(EBOV-GP) using an m.o.i. of 0.0001 ffu cell^−1^. Spreading of virus in the cell monolayer was monitored by detection of GFP fluorescence with an inverted fluorescence microscope. The bar represents 200 µm. (c) Multi-cycle replication of sNLuc reporter viruses in IFN-competent cells. NHDF were infected with either VSV∆G(EBOV-GP,sNLuc) (red line) or VSVMq∆G(EBOV-GP,sNLuc) (black lines) using an m.o.i. of either 0.001 ffu cell^−1^ (circles), 0.01 ffu cell^−1^ (rhombs) or 0.1 ffu cell^−1^ (squares). sNLuc activity was determined in the cell-culture medium at the indicated times. Mean luciferase values and sd of three infection experiments are shown. Asterisks indicate significant different luciferase values compared to VSV∆G(EBOV-GP,sNLuc).

In order to investigate the induction of type I IFN or other antiviral cytokines by the vaccine candidates, normal human dermal fibroblasts (NHDF) were infected with the recombinant viruses using an m.o.i. of 1 ffu cell^−1^. At 24 h p.i., cell-culture supernatants were collected and heated for 30 min at 55 °C to inactivate any infectious virus [23]. The cell-culture supernatants were serially diluted and subsequently incubated with HeLa cells for 24 h. Finally, the induction of an antiviral state in the HeLa cells was determined with a bioassay taking advantage of VSV*ΔG(Luc) replicon particles encoding the firefly luciferase reporter protein [24]. It turned out that all viruses that expressed wild-type M protein completely suppressed the synthesis of antiviral cytokines, whereas the infection of NHDF with VSV*Mq or VSV*MqΔG(EBOV-GP) led to a strong induction of type I IFN (Fig. 4a). Accordingly, the dissemination of VSV*MqΔG(EBOV-GP) was severely restricted in NHDF but was not affected in Vero cells that are unable to produce type I IFN (Fig. 4b). In contrast, VSV*ΔG(EBOV-GP) expressing wild-type VSV-M protein showed spreading in both NHDF and Vero cells, although dissemination was much slower in NHDF compared to Vero cells. The replication of M protein-modified VSV was also studied using sNLuc reporter viruses. Following infection of NHDF with VSVMqΔG(EBOV-GP,sNLuc) using an m.o.i. of 0.001 ffu cell^−1^, sNLuc expression levels were suppressed approximately 100-fold compared to cells infected with VSVΔG(EBOV-GP,sNLuc) (Fig. 4c). However, attenuation of VSVMqΔG(EBOV-GP,sNLuc) was compensated if the cells were infected with a higher virus dose. Using an m.o.i. of 0.1 ffu cell^−1^, the luciferase reporter reached levels at 36 and 48 h p.i. that were similarly high as those produced by a 100-fold lower dose of VSVΔG(EBOV-GP,sNLuc).

### Analysis of vector-driven EBOV-GP expression

**Fig. 5.**
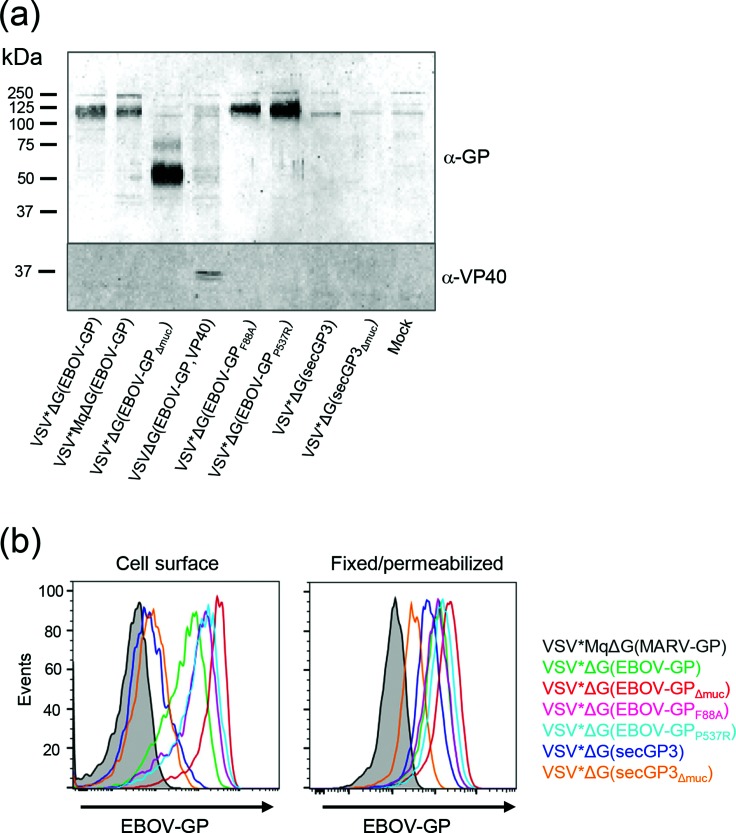
Recombinant VSV-driven expression of EBOV-GP. Vero cells in 12-well plates were infected with the indicated viruses using an m.o.i. of 10 ffu cell^−1^. (a) At 14 h p.i., cell surface proteins were labelled with biotin, precipitated from cell lysates by immobilized streptavidin, and analysed by Western blot using either guinea pig polyclonal anti-EBOV-GP serum or rabbit anti-VP40 serum. The positions of proteins with defined molecular weight are indicated on the left-hand side. (b) Flow cytometric analysis of EBOV-GP expression. Infected Vero cells were incubated with a LIVE/DEAD fixable violet dead cell marker. Subsequently, cells were stained either directly for EBOV-GP cell surface expression (left panel) or fixed and permeabilized to allow detection of intracellular EBOV-GP (right panel). EBOV-GP was detected using a mouse polyclonal anti-EBOV-GP anti-serum and anti-mouse IgG-allophycocyanin. Expression levels of EBOV-GP are represented in histogram plots of live, GFP-positive, i.e. infected cells.

For detection of mature EBOV-GP at the cell surface, VSV vector-infected cells were labelled with sulfo-NHS-LC-LC-biotin. The biotinylated cell surface proteins were precipitated from cell lysates with immobilized streptavidin, separated by SDS-PAGE under reducing conditions and analysed by Western blot using guinea pig anti-EBOV-GP serum. At 14 h p.i. of Vero cells with either VSV*ΔG(EBOV-GP) or VSV*MqΔG(EBOV-GP), GP migrating as a single band of 120 kDa was detected at the cell surface (Fig. 5a). In contrast, EBOV-GP_Δmuc_ lacking the heavily O-glycosylated mucin-like domain showed a drastically reduced apparent molecular weight of about 55 kDa. Infection of cells with VSVΔG(EBOV-GP,VP40) resulted in very low EBOV-GP expression levels at the cell surface at 14 h p.i., whereas EBOV-GP_F88A_ and EBOV-GP_P537R_ were well expressed. As expected, the soluble EBOV glycoproteins secGP3 and secGP3∆muc were not found at the cell surface. The EBOV matrix protein VP40 was exclusively detected in lysates of cells that had been infected with VSVΔG(EBOV-GP,VP40).

Cell surface expression of recombinant EBOV-GP was also analysed by flow cytometry using mouse polyclonal anti-EBOV-GP serum. Using this approach, the mutant glycoproteins EBOV-GP_F88A_ and EBOV-GP_P537R_ and, in particular, EBOV-GP_Δmuc_ were detected at the cell surface at higher levels than the wild-type glycoprotein (Fig. 5b, left panel). The anti-EBOV mouse serum also reacted weakly with Vero cells infected with VSV*ΔG(secGP3) and VSV*ΔG(secGP3_Δmuc_). This signal might be due to secreted EBOV-GP which stayed associated with the cell surface. A clear signal was detected for secGP3 and secGP3_Δmuc_ by intracellular staining assay (Fig. 5b, right panel), confirming that both secGP3 and secGP3_Δmuc_ were actually expressed by the infected cells.

### Analysis of immune sera from vaccinated guinea pigs

**Fig. 6.**
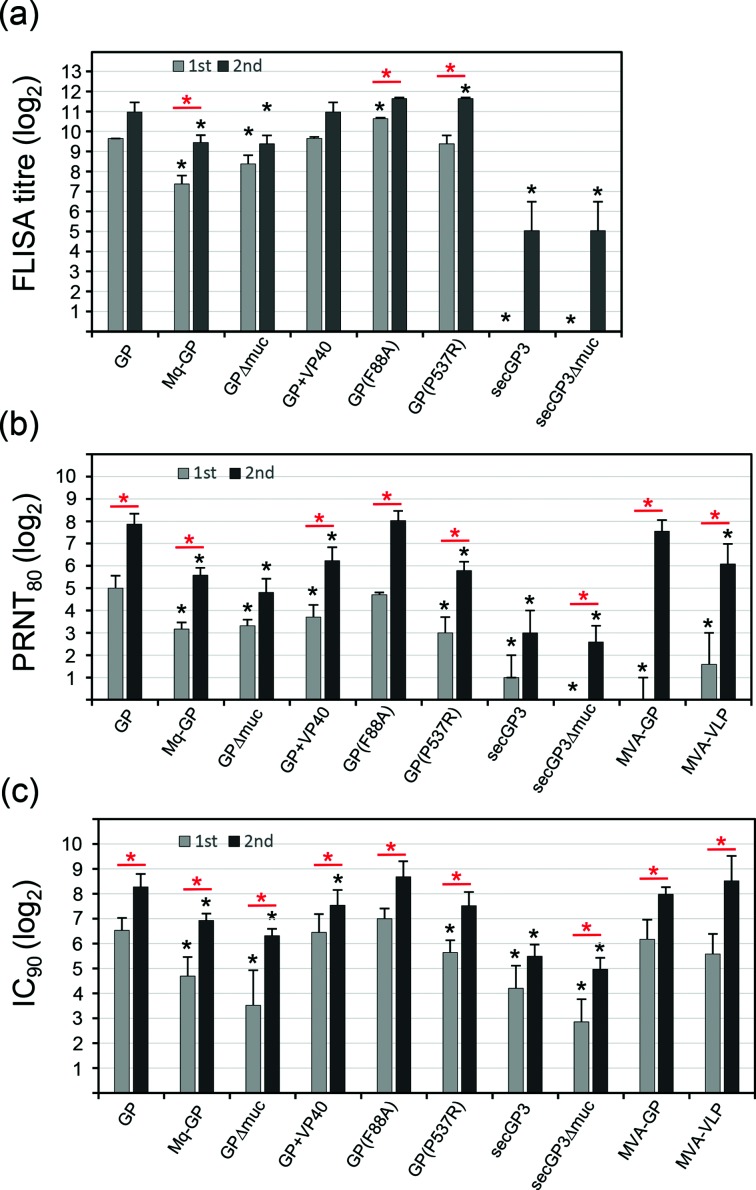
Analysis of the antibody responses of guinea pigs vaccinated with recombinant VSV. (a) FLISA. Vero cells were grown in 96-well plates and infected with MVA-BN-EBOV-GP (m.o.i. of 0.05 ffu cell^−1^). At 24 h p.i., the cells were fixed with paraformaldehyde and incubated with serially diluted serum pools from vaccinated guinea pigs (four to five animals per vaccine group) and subsequently with Alexa 488-conjugated anti-guinea pig IgG serum. FLISA antibody titres were calculated by determining the reciprocal value of the highest immune serum dilution allowing discrimination of infected from non-infected Vero cells by indirect immunofluorescence. Mean values and sd of three independently performed titrations are shown. (b) Analysis of guinea pig sera by PRNT. Serially diluted serum from vaccinated guinea pigs (*n*=4 to 5 animals per vaccine group) were incubated for 60 min with 100 ffu of VSV*∆G(EBOV-GP). Vero cell monolayers grown in 96-well cell-culture plates were inoculated with the virus/antibody mixture for 1 h and then replaced by 200 µl of medium containing 0.8 % methyl cellulose. Following an incubation period of 24 h, the GFP-positive cell foci were counted under an inverted fluorescence microscope. The reciprocal serum dilution causing a reduction of plaque numbers by 80 % (PRNT_80_) was calculated. Mean titres and sd were calculated for the immune sera collected from four to five individual guinea pigs per group. (c) Inhibition of virus spreading *in vitro*. Vero cells were infected with VSV*∆G(EBOV-GP,sNLuc) using an m.o.i. of 0.005 ffu cell^−1^ and maintained in medium containing serial dilutions of immune sera which were collected 4 weeks after the first and 4 weeks after the second immunization of guinea pigs with recombinant VSV expressing the indicated antigens. At 24 h p.i., sNLuc activity in the cell-culture supernatant was determined. The reciprocal serum dilution leading to 90 % inhibition of reporter activity (IC_90_) was determined (relative to virus-spreading experiments in the presence of naïve guinea pig serum). Mean IC_90_ titres and sd were calculated for immune sera that were collected from four to five individual guinea pigs per vaccine group. (a–c) Black asterisks indicate significantly different titres (*P*<0.05) with respect to the reference vaccine VSV*∆G(EBOV-GP). Red asterisks indicate significantly different antibody titres when comparing first and second immunization.

Guinea pigs were immunized via the intramuscular route with the recombinant vector vaccines (10^8^ ffu per animal), which had been produced on helper cells providing the VSV-G protein *in trans*, and blood was collected 4 weeks after the primary immunization. Subsequently, the animals were immunized a second time with the same vector and dose, and sera were prepared 4 weeks later. The immune sera were titrated by taking advantage of a FLISA that was based on MVA-BN-EBOV-GP-infected Vero cells (Fig. 6a). Four weeks after the primary immunization with our reference vaccine VSV*∆G(EBOV-GP), immune serum revealed an EBOV-GP-specific antibody titre of 800 (a dilution of 1 : 800 was still able to discriminate between MVA-BN-EBOV-GP infected and non-infected cells). Four weeks after the second immunization, the antibody titre had increased, but this increase was rather small and could not be tested as significantly different. Likewise, small and non-significant increases of antibody titres were found following the second immunization with VSV*∆G(EBOV-GP_∆muc_), VSV∆G(EBOV-GP,VP40), VSV*ΔG(EBOV-sGP3) and VSV*ΔG(EBOV-sGP3_Δmuc_), whereas a significant boosting effect was observed with VSV*MqΔG(EBOV-GP), VSV*∆G(EBOV-GP_F88A_) and VSV*∆G(EBOV-GP_P537_). The first immunization with VSV∆G(EBOV-GP,VP40) and VSV*∆G(EBOV-GP_P537_) led to antibody titres that were as high as those induced by the reference vaccine, while VSV*∆G(EBOV-GP_F88A_) induced significantly higher titres. In contrast, following the first immunization with VSV*Mq∆G(EBOV-GP) and VSV*∆G(EBOV-GP_∆muc_) antibody titres were significantly lower than those induced by the reference vaccine, while vaccination with soluble antigen (secGP3 or secGP3_∆muc_) did not result in detectable antibody titres. Following the second immunization with VSV∆G(EBOV-GP,VP40) or VSV*∆G(EBOV-GP_F88A_), antibody titres were as high as those induced by the reference vaccine (second immunization), while VSV*∆G(EBOV-GP_P537_) induced a significantly higher titre. In contrast, the antibody titres induced by the second application of VSV*Mq∆G(EBOV-GP) or VSV*∆G(EBOV-GP_∆muc_) were significantly lower than those induced by VSV*∆G(EBOV-GP). These findings suggest that VSV*Mq∆G(EBOV-GP) and VSV*∆G(EBOV-GP_∆muc_) are less immunogenic than the reference virus VSV*∆G(EBOV-GP). The vaccine constructs VSV*ΔG(EBOV-sGP3) and VSV*ΔG(EBOV-sGP3_Δmuc_) seemed to be nonimmunogenic, at least with regard to the induction of antibodies that would bind to native EBOV-GP antigen.

In order to functionally characterize guinea pig immune sera, a PRNT was performed with VSV*∆G(EBOV-GP) as a surrogate virus. This assay was chosen because recent work suggested a high degree of correlation between BSL-2 pseudotyped VSV fluorescence reduction neutralization test and BSL-4 EBOV neutralization assays [25]. We used an 80 % PRNT (PRNT_80_), as the sensitivity of the test was not sufficiently high to capture the 90 % reduction values for the primary immunization sera. The immune sera from the vaccine groups VSV*∆G(EBOV-GP), VSV*Mq∆G(EBOV-GP), VSV*∆G(EBOV-GP_∆muc_), VSV*∆G(EBOV-GP_F88A_, VSV*ΔG(EBOV-sGP3) and VSV*ΔG(EBOV-sGP3_Δmuc_) revealed PRNT_80_titres (Fig. 6b), which followed a very similar pattern as seen before with FLISA (Fig. 6a). However, animals immunized with either VSV*∆G(EBOV-GP_P537_) or VSV∆G(EBOV-GP,VP40) produced significantly lower PRNT_80_ titres than animals that had received the reference vaccine. Since all three vaccines induced similar antigen-binding antibody titres (Fig. 6a), the quality of the antibodies induced by the different vaccine constructs likely differed. In contrast to FLISA, all vaccine constructs except VSV*∆G(EBOV-GP_∆muc_) revealed significantly increased PRNT_80_ titres after the second immunization, indicating that the quality of the neutralizing antibodies was benefiting from the second immunization while having only a moderate effect on antigen-binding antibody levels. We also analysed immune sera from guinea pigs that had been immunized with recombinant MVA [26], a viral vector which is propagation-incompetent in most mammalian cells [27, 28]. Four weeks after the primary immunization with a high dose (5×10^8^ ffu) of either MVA-BN-EBOV-GP (expressing EBOV-GP) or MVA-BN-EBOV-VLP (expressing EBOV-GP, EBOV-VP40 and TAFV-NP), PRNT_80_ titres were not detectable or very low (Fig. 6b). Only after the second immunization, antibody titres increased significantly reaching mean values of 188 and 68, respectively.

Since antibodies may not only interfere with receptor-binding and fusion but also with virus-budding and release [29], we wondered whether a virus-spreading inhibition test might be more sensitive than the PRNT_80_ test. To test this hypothesis, Vero cells were infected with VSV*ΔG(EBOV-GP,sNLuc), a chimeric VSV expressing the sNLuc reporter protein, using an m.o.i. of 0.001 ffu cell^−1^, and subsequently incubated with medium containing guinea pig anti-EBOV-GP immune serum. At 24 h p.i., sNLuc activity in the cell-culture supernatant was determined and results expressed as the inhibitory concentration suppressing the spreading of VSV*ΔG(EBOV-GP,sNLuc) by 90 % (IC_90_) (Fig. 6c). It turned out that the IC_90_titres for most vaccine groups showed a similar pattern as the PRNT_80_ titres although the absolute values were generally higher. In contrast to PRNT, however, immune sera from animals immunized once with VSV∆G(EBOV-GP,VP40) revealed inhibitory antibody titres that were as high as those induced by the reference vaccine. There were also other discrepancies observed when the relative PRNT_80_ and IC_90_ titres were compared. Immune sera that were prepared following the second immunization with VSV*∆G(EBOV-GP_P537_) showed IC_90_ titres that were as high as those induced by the reference vaccine, although PRNT_80_ titres were significantly different between the two vaccine groups. Furthermore, a single immunization with either MVA-BN-EBOV-GP or MVA-BN-EBOV-VLP induced similar IC_90_ titres as VSV*∆G(EBOV-GP), although the corresponding PRNT_80_ titres were low or undetectable (Fig. 6b). These discrepancies between PRNT80 and IC90 titres might be at least partially attributed to the higher sensitivity of the virus-spreading inhibition test. It should also be noted that the virus-spreading inhibition test would also gather EBOV-GP-specific antibodies that interfere with virus-budding and release.

## Discussion

The generation of live-attenuated virus vaccines has always been a great challenge irrespective of whether these vaccines have been generated by recombinant DNA technologies or by classical means (e.g. serial passaging the virus on different host cell lines). As attenuation of viruses is frequently accompanied with loss in immunogenicity, a major difficulty in generating live-attenuated virus vaccines is to find an adequate balance between sufficient attenuation (safety) and maintenance of immunogenicity. The recently developed Ebola vaccine candidate VSV/EBOV-GP is a modified VSV in which the VSV-G gene has been replaced by the EBOV glycoprotein gene [14]. VSV/EBOV-GP turned out to be highly attenuated as intracerebral inoculation of non-human primates with this virus did not result in apparent disease [9, 10]. It was therefore surprising to see that the vaccine caused adverse effects such as fever and arthritis in human volunteers who were enrolled in a clinical phase 2 study in Geneva, Switzerland [11–13]. These unwanted complications might be related to the ability of the chimeric virus to still cause viremia in humans [30], a feature which has also been observed in non-human primates [4]. The aim of our study was therefore to develop a highly attenuated VSV-based vector vaccine with a compromised ability of spreading.

Our first approach of attenuation was based on a VSV vector encoding a modified VSV-M protein (Mq), which is known to be free of host shut-off activity [18]. As expected, infection of NHDF with VSV*MqΔG(EBOV-GP) led to the synthesis and secretion of type I IFN. Due to the paracrine action of this antiviral cytokine, the virus was unable to spread in cell culture beyond the primary infected cells. In addition, the autocrine action of type I IFN resulted in suppression of virus replication and lower reporter protein levels (see Fig. 4). The lower antigen levels and reduced spreading in tissues might explain why immunization with this vaccine induced lower levels of neutralizing antibodies compared to a vaccine vector which expressed the wild-type M protein. However, when VSV*MqΔG-EBOV-GP was applied a second time, antibody titres were significantly boosted. In line with this observation, others have shown that VSV-vectored vaccines that express a modified M protein are sufficiently immunogenic [31] and can protect animals from infection with vaccinia virus or VSV [32, 33]. Future experiments will show whether VSV*MqΔG(EBOV-GP) is able to protect non-human primates from EBOV challenge infection. As Mq-modified VSV is restricted in spreading and probably unable to cause viremia, it represents a safer vector vaccine than the unmodified VSV vector. Of note, Mq-modified VSV vectors can be propagated to high titres on Vero cells which have a defect in the synthesis of type I IFN.

Another strategy of attenuation was based on a modified EBOV-GP antigen in which the large mucin-like domain had been deleted. This modification did not affect propagation of VSV*ΔG(EBOV-GP_∆muc_) on Vero cells, indicating that this domain is not essential for viral replication *in vitro*. VSV*ΔG(EBOV-GP_∆muc_) replicated even faster than VSV*ΔG-EBOV-GP, a phenomenon that may be attributed to the shorter genome of VSV*ΔG(EBOV-GP_∆muc_) differing from the parental VSV*ΔG(EBOV-GP) genome by 570 nucleotides. Although the modified antigen was expressed at the cell surface at high levels, it induced significantly less neutralizing antibodies in vaccinated guinea pigs than the authentic EBOV-GP antigen, indicating that the deletion of the mucin-like domain had a negative impact on the immunogenic properties of the viral glycoprotein. Indeed, a number of known protective antibodies such as 6D8, 13F6 and 13C6 have been shown to bind to the mucin-like domain [34, 35].

VSVΔG(EBOV-GP,VP40) expressing both EBOV-GP and EBOV-VP40 turned out to be significantly attenuated on Vero cells, showing slower replication kinetics and reaching lower final virus titres than the reference vaccine VSV*ΔG(EBOV-GP). Correspondingly, EBOV-GP expression levels at the cell surface were also quite low early in infection. Nevertheless, immunization of guinea pigs with VSVΔG(EBOV-GP,VP40) resulted in antigen-binding antibody titres that were comparable to those induced by VSV*ΔG(EBOV-GP). However, neutralizing activity of these antibodies was significantly lower than those induced by VSV*ΔG(EBOV-GP). This attenuation has not been observed with the MVA-BN-EBOV-VLP vector expressing the same two EBOV antigens [26]. It is therefore possible that the VP40 protein had a specific negative impact on VSV replication/transcription or VSV matrix protein-mediated budding. However, work with a similar VSV vaccine construct based on SUDV-GP and SUDV-VP40 suggested that antibody titres stimulated by a homologous prime/boost regimen might be sufficiently high to provide protection of non-human primates [17].

There is evidence that adenovirus-vectored and MVA-vectored Ebola vaccines can induce protective immune responses in human volunteers even though these vectors are propagation-incompetent [36]. This observation stimulated us to generate a propagation-incompetent VSV-vectored EBOV vaccine by expressing soluble versions of the EBOV-GP protein. As these glycoproteins lacked the transmembrane domain, they could not take part in the process of virus budding. These vector vaccines were propagated to high titres on helper cells that expressed the VSV-G protein in a regulated manner [37]. Nevertheless, immunization of guinea pigs with these vaccines resulted in only low titres of neutralizing antibodies. Indeed, the multivalent interaction of cognate B cell receptors with multiple GP spikes presented on the viral envelope or the surface of GP-expressing cells may particularly be important to induce conformation-dependent neutralizing antibodies. This hypothesis is supported by the observation that secreted GP, which is produced at large amounts by EBOV-infected cells as a result of RNA editing [38, 39], induces only low levels of neutralizing antibodies but serves as a decoy antigen which snatches neutralizing antibodies away [40, 41].

As an alternative approach for the generation of propagation-restricted VSV-vectored Ebola vaccine, we expressed mutant EBOV-GP known to be functionally defective. The F88A mutation has been reported to render EBOV-GP defective for mediating virus entry into a variety of human cell types, including antigen-presenting cells [19]. The P537R mutation, which is located close to the putative fusion domain of the glycoprotein, was shown to compromise virus entry although it did not affect EBOV-GP transport to the cell surface or its incorporation into the viral envelope [21]. In line with these reports, VSV*ΔG(EBOV-GP_F88A_) and VSV*ΔG(EBOV-GP_P537R_) did not replicate on Vero cells but could be propagated on helper cells expressing VSV-G protein. In the absence of VSV-G protein expression, VSV*ΔG(EBOV-GP_P537R_) and VSV*ΔG(EBOV-GP_F88A_) propagated on BHK-G43 cells to low titres suggesting that infectivity of these mutant viruses is significantly reduced but not completely abolished. The attenuated phenotype was not stably maintained when the mutant viruses were passaged since both VSV*ΔG(EBOV-GP_F88A_) and VSV*ΔG(EBOV-GP_P537R_) rapidly acquired compensating mutations that led to higher infectious virus titres. This finding underscores the extraordinary plasticity of RNA viruses. It may be speculated that the VSV/EBOV-GP vaccine that was employed in the Geneva clinical phase study [12, 30] may have undergone mutational changes as well, in particular when volunteers received a high vaccine dose. Any mutation that may have allowed higher virus replication rates could have been responsible for enhanced virus dissemination, leading to the observed adverse effects. Unfortunately, VSV/EBOV-GP has not been isolated from volunteers post vaccination and therefore corresponding cDNA sequences are not available.

Immunization of guinea pigs with VSV*ΔG(EBOV-GP_F88A_) triggered the production of neutralizing antibodies at levels that were as high as those induced by immunization with the propagation-competent VSV*ΔG-EBOV-GP vaccine. In contrast, VSV*ΔG(EBOV-GP_P537R_) induced lower titres of neutralizing antibodies suggesting that the P537R mutation had a negative effect on the immunogenicity of the antigen. Thus, VSV*ΔG(EBOV-GP_F88A_) may be selected for further vaccine development. First, it will be necessary to introduce additional mutations into the GP gene that interfere with virus entry in order to make emergence of revertant viruses more unlikely and the vaccine candidate genetically more stable.

In conclusion, propagation-restricted VSV vectors may represent a safe alternative to propagation-competent VSV vectors. In a preventative vaccination scenario, propagation-restricted VSV vectors might be preferentially used in heterologous prime-boost protocols in combination with MVA- or adenovirus-vectored EBOV vaccines [42–44]. Finally, propagation-restricted VSV vectors may be used for the development of safe vector vaccines for protection against other zoonotic pathogens.

## Methods

### Cells

Vero cells (C1008) were purchased from the American Type Culture Collection (ATCC; Manassas, VA, USA) and maintained in Glasgow’s minimal essential medium (GMEM; Life Technologies) supplemented with 5 % fetal bovine serum (FBS). BHK-G43, a transgenic BHK-21 cell clone expressing the VSV-G protein in a regulated manner [37], was maintained in GMEM containing 5 % FBS. HeLa (ATCC) and normal human dermal fibroblasts (NHDF; Lonza, Basel, Switzerland) were maintained in Eagle’s minimal essential medium (EMEM) supplemented with 10 % FBS. The UMNSAH/DF-1 (DF-1) chicken fibroblast cell line (ATCC) was maintained in Dulbecco’s modified Eagle’s medium and 10 % FBS. All cell lines were cultured at 37 °C in a humidified atmosphere containing 5 % CO_2_, except DF-1 cells which were kept at 39 °C.

### Virus

Recombinant VSV* expressing GFP from an extra transcription unit and VSV*Mq expressing a mutant M protein with four distinct point mutations (Mq) have been described previously [18]. Propagation-incompetent VSV*∆G(Luc) lacking the G gene and expressing both GFP and firefly luciferase (Luc) was produced and propagated as previously described [24]. The recombinant viruses VSV*Mq∆G(EBOV-GP), MVA-BN-EBOV-GP and MVA-BN-EBOV-VLP have recently been described [26]. Recombinant MVA-T7 expressing the T7 RNA polymerase was kindly provided by Gerd Sutter (LMU München, Germany). Recombinant MVA-BN-EBOV-GP and MVA-BN-EBOV-VLP were propagated on primary chicken embryo fibroblasts and MVA-T7 on DF-1 cells.

### Generation of recombinant plasmids

All plasmids were based on the previously published plasmid pVSV*ΔG(HA) encoding a modified VSV (serotype Indiana) genome with six transcription units [45]. The fourth transcription unit of this plasmid harbored the influenza virus HA gene (flanked by endonuclease restriction sites *Mlu*I and *Bst*EII) while the fifth transcription unit contained the enhanced GFP gene flanked by *Xho*I and *Nhe*I restriction sites. The genomic plasmid pVSV*ΔG(EBOV-GP) was generated by replacing the HA gene with a synthetic codon-optimized EBOV-GP gene (accession number NP_066246) taking advantage of the *Mlu*I and *Bst*EII endonuclease restriction sites. By taking advantage of the *Xho*I and *Nhe*I endonuclease restriction sites, the GFP gene in pVSV*ΔG(EBOV-GP) was replaced with the sNLuc gene (Promega, Madison, Wisconsin, USA) resulting in pVSVΔG(EBOV-GP,sNLuc). We employed the same strategy to substitute the GFP gene of pVSV*ΔG(EBOV-GP) with the EBOV VP40 gene (strain Mayinga, accession number: AF086833; kindly provided by Elke Mühlberger, Boston University, MA, USA), which resulted in the recombinant plasmid pVSVΔG(EBOV-GP,VP40). The plasmid pVSV*MqΔG(EBOV-GP) containing the Mq gene with the mutations M33A, M51R, V221F and S226R [18] was generated by replacing the *Xba*I/*Mlu*I region of pVSV*ΔG(EBOV-GP) with the corresponding fragment from pVSV*Mq [18]. EBOV-GP with either the point mutation F88A or P537R or the deletion of the mucin-like domain (amino acids 330–489) were generated by emplyoing overlapping PCR technology. The mutant EBOV-GP genes then replaced the wild-type EBOV-GP gene in pVSV*ΔG(EBOV-GP), resulting in plasmids pVSV*ΔG(EBOV-GP_F88A_), pVSV*ΔG(EBOV-GP_P537R_) and pVSV*ΔG(EBOV-GP_Δmuc_), respectively. A soluble trimeric EBOV-GP was generated by fusing the cDNA encoding the EBOV-GP amino acids 1–643 to the nucleotide sequence encoding the GCN4-pII trimeric coiled coil domain plus a stop codon (ATGAAACAGATCGAGGATAAGATCGAGGAAATTCTGAGCAAGATCTATCACATTGAAAACGA AATCGCAAGAATCAAGAAACTGGTGGGGGAAAGATGA). The resulting secGP3 gene replaced the EBOV-GP gene in pVSV*ΔG(EBOV-GP) resulting in pVSV*ΔG(secGP3). The genomic plasmid pVSV*ΔG(secGP3_Δmuc_) was produced correspondingly using the EBOV-GP_Δmuc_ gene as a template for amplification of the insert by PCR.

### Generation and titration of recombinant VSV

Recombinant VSV vectors were generated on VSV-G protein expressing BHK-G43 helper cells as described previously [24]. The viruses were titrated in duplicate on Vero cells grown in 96-well microtitre plates. The confluent cell monolayers were inoculated (40 µl well^−1^) with 10-fold dilutions of each virus for 90 min at 37 °C and overlaid with 160 µl well^−1^ of GMEM containing 2 % fetal calf serum and 0.9 % methylcellulose (Sigma). At 20 h p.i., the GFP-positive cell foci were counted using an inverted fluorescence microscope and the infectious virus titre calculated and expressed as ffu ml^−1^.

For detection of cells that had been infected with chimeric VSV lacking the GFP reporter, e.g. VSVΔG(EBOV-GP,VP40) or VSVΔG(EBOV-GP,sNLuc), the cells were washed twice with PBS at 20 h p.i. and fixed with 3 % paraformaldehyde for 30 min at room temperature. Excess paraformaldehyde was quenched with 0.1 M glycine in PBS for 5 min. The cells were permeabilized with 0.25 % Triton X-100 in PBS for 5 min at room temperature and incubated for 60 min at room temperature with a monoclonal antibody (1 : 40 in PBS) directed to the VSV matrix protein (hybridoma clone 23H12; Kerafast, Boston, USA). The cells were washed three times with PBS and incubated for 1 h at room temperature with Alexa Fluor 488-conjugated goat anti-mouse IgG serum (4 µg ml^−1^, 100 µl well^−1^). After three wash steps, infected cells were detected with an inverse fluorescence microscope.

### Virus replication kinetics

Multi-step replication of recombinant VSV was analysed using Vero cells. Confluent cell monolayers seeded the day before in six-well cell-culture plates were inoculated with virus at 37 °C for 1 h using an m.o.i. of 0.0001 ffu cell^−1^. Three wells were infected in parallel with each virus. After adsorption, the inoculum was removed and the cells were washed three times with 5 ml of GMEM before addition of 2.5 ml of GMEM containing 5 % FBS. At the indicated times, aliquots of 250 µl were taken and replaced by the same volume of fresh medium. The aliquots were stored frozen at −70 °C until titration (see above).

### Serial passaging of recombinant VSV encoding mutant EBOV-GP

BHK-21 cells (grown in six-well plates) were infected (m.o.i. of 1 ffu cell^−1^) with either VSV*ΔG(EBOV-GP_F88A_) or VSV*ΔG(EBOV-GP_P537R_) that have been produced on BHK-G43 helper cells. Following infection, the cells were maintained for 24 h in the presence of neutralizing anti-VSV-G antibody (hybridoma clone I1, ATCC). The cell-culture supernatants were harvested and stored frozen in aliquots at −70 °C. The viruses were diluted 1 : 2 and subsequently passaged for five times on BHK-21, each passage performed in six parallel wells. Virus titres were determined for each passage as described above. Total RNA was extracted from BHK-21 cells that have been infected with passage 5 viruses that turned out to produce increased infectious titres. The RNA was reversed transcribed with Superscript III reverse transcriptase (Thermo Fisher Scientific) and random hexamers for priming. The EBOV-GP cDNA was amplified by PCR with Phusion Hot Start II DNA Polymerase (Thermo Fisher Scientific) and inserted into the pJet1.2 plasmid (Thermo Fisher Scientific). *E. coli* were transformed with the recombinant plasmids and selected on LB agar plates containing ampicillin. The complete ORF of the cloned GP isolated from three bacterial colonies were sequenced using BigDye Terminator v3.1 Cycle Sequencing Kit (Life Technologies) and an Applied Biosystems 3130 automated Genetic Analyzer (Applied Biosystems).

### Immunization of guinea pigs

Dunkin-Hartley guinea pigs were provided by the animal breeding facility of the Institute of Virology and Immunology (IVI) in Mittelhäusern, Switzerland. Animals with a weight of 400 to 500 grams were immunized intramuscularly by injection of 250 µl of GMEM containing 2×10^8^ ffu ml^−1^ of recombinant VSV (propagated on BHK-G43 helper cells) or 5×10^8^ TCID_50_ of recombinant MVA into the femoral muscle of each hind leg. After 4 weeks, 2 ml of blood was collected from each animal under anesthesia by heart puncture. The animals were immunized a second time using the same vector vaccine, route and dosage. Four weeks after the second immunization, the guinea pigs were bled under anesthesia. Sera were prepared by centrifugation of coagulated blood and stored in aliquots at −20 °C.

### Fluorescence-linked immunosorbent assay

Vero cells were grown for 24 h in 96-well microtitre plates and infected with MVA-BN-EBOV-GP using an m.o.i. of 0.05 ffu cell^−1^. At 24 h p.i., the cells were fixed with 3 % paraformaldehyde in PBS for 30 min at room temperature and subsequently washed two times with PBS containing 0.1 M glycine and once with PBS. The guinea pig immune sera were serially diluted in PBS and incubated for 60 min at room temperature with the fixed cells (100 µl well^−1^). The cells were washed three times with PBS (250 µl well^−1^) and subsequently incubated for 60 min at room temperature in the dark with Alexa Fluor 488-conjugated goat anti-guinea pig IgG serum (4 µg ml^−1^, 100 µl well^−1^). Finally, the cells were washed three times as above and then investigated by fluorescence microscopy (AxioVert 2, Zeiss, Jena, Germany). The antibody titre was determined by calculating the reciprocal value of the highest immune serum dilution allowing discrimination of infected from non-infected Vero cells. The titration was performed three times and mean values and sd were calculated.

### Plaque reduction neutralization assay

Serial twofold dilutions of guinea pig immune sera were incubated in quadruplicates for 1 h at 37 °C with 100 ffu of VSV*Mq∆G(EBOV-GP), which has been propagated on Vero cells, and then added to Vero cell monolayers grown in 96-well cell-culture plates. After an incubation period of 1 h at 37 °C, the inoculum was removed and 200 µl of GMEM containing 2 % FBS and 0.8 % methyl cellulose (Sigma-Aldrich; Buchs, Switzerland) were added. Following an incubation period of 24 h at 37 °C, the GFP-positive cell foci were counted under an AxioVert inverted fluorescence microscope. The reciprocal serum dilution causing a reduction of plaque numbers by 80 % (PRNT_80_) was calculated.

### Inhibition of virus spread *in vitro*

Vero cells grown in 96-well tissue culture plates (2×10^4^ cells/well) were infected for 1 h at 37 °C with 50 µl per well containing 100 ffu of Vero cell-grown VSV* ∆G(EBOV-GP,sNLuc). The cells were washed once with GMEM and incubated 24 h at 37 °C with 100 µl well^−1^ of GMEM containing 5 % FBS and serially diluted immune sera. To determine sNLuc activity, 25 µl of the cell-culture supernatant was transferred to a black 96-well microtitre plate and 25 µl of Nano-Glo luciferase substrate (Promega, Madison, Wisconsin, USA) was added to each well. Luminescence was recorded for 1 s with a Centro LB 960 luminometer (Berthold Technologies, Bad Wildbad, Germany). The reciprocal immune serum dilution leading to 90 % inhibition of sNLuc activity (relative to virus spreading in the presence of naïve guinea pig serum) was calculated and expressed as IC_90_.

### IFN bioassay

NHDF were grown in 24-well cell-culture plates and infected with recombinant VSV (m.o.i. of 3 ffu cell^−1^). The cell-culture supernatants were collected 24 h p.i. and any virus was inactivated by heating the supernatants for 30 min at 55 °C [23]. The concentration of secreted type I IFN was determined by titrating the conditioned medium on HeLa cells as previously described [24]. The dilution of conditioned medium causing 50 % suppression of VSV*∆G(Luc)-driven luciferase expression was calculated and expressed as inhibitory concentration 50 % (IC_50_).

### Flow cytometry

Single-cell suspensions of infected cell monolayers were prepared by scraping and thoroughly suspending the cells in culture medium. Cells were washed with ice-cold PBS and stained with a LIVE/DEAD fixable violet dead cell staining kit according to the manufacturer’s instructions (Thermo Fisher Scientific, Bonn, Germany) to exclude dead cells during analysis. Cells were then suspended in ice-cold PBS containing 2 % FCS and either fixed/permeabilized with Fixation/Permeabilization solution kit (BD Biosciences, Heidelberg, Germany) or left untreated. Fixed/permeabilized and non-permeabilized cells were stained using a pool of polyclonal mouse serum (1 : 1000) obtained by immunization of C57BL/6 mice with MVA-BN-EBOV-GP or MVA-BN-EBOV-VLP, followed by staining with an allophycocyanin-coupled anti-rabbit secondary antibody (1 : 1000). Cells were analysed for viability, intracellular GFP expression as a marker of infection as well as for surface and cytoplasmic expression of EBOV-GP by flow cytometry using a LSR II flow cytometer (BD Biosciences, Heidelberg, Germany) and FlowJo software (Tree Star, Ashland, OR, USA).

### Cell surface biotinylation

Vero cells grown in six-well plates were infected with recombinant VSV using an m.o.i. of 10 ffu cell^−1^. At 14 h p.i., cell surface proteins were labelled at 4 °C with sulfo-NHS-LC-LC-biotin (Life Technologies Europe, Zug, Switzerland) and precipitated from cell lysates with streptavidin-agarose (Life Technologies Europe) as described previously [46]. The precipitated cell surface proteins were run on SDS-PAGE (10 %) under reducing conditions and analysed by Western blot using polyclonal anti-EBOV-GP serum (1 : 4000) from MVA-BN-EBOV-GP vaccinated guinea pigs.

### Statistical analysis

Mean values and sd were calculated. Data were analysed by Student's *t*-test and *P*<0.05 was considered significant.
